# Pre‐ and Postvaccination Effects on Milk Yield and Quality Among Dairy Farms and Lactating Cows in Mekelle, Ethiopia: An Ambispective Study

**DOI:** 10.1155/vmi/2417101

**Published:** 2026-07-19

**Authors:** Tadelech Yilma Sisay, Mastewal Addisu Kitambo, Kidist Derbew Gebru, Mebrahtu Weldegebriel Hailu, Haftom Yirga Tsegay

**Affiliations:** ^1^ College of Veterinary Medicine and Animal Sciences, Mekelle University, Mekelle, Tigray, Ethiopia, mu.edu.et

**Keywords:** dairy, farm, milk-quality, milk-yield, vaccine-coverage

## Abstract

**Background:**

Vaccination plays a critical role in improving animal health and productivity; however, its impact on milk yield and quality under field conditions remains insufficiently quantified in Ethiopia.

**Aims:**

This study aimed to evaluate the pre‐ and postvaccination effects on milk yield and quality among medium‐dairy farms and lactating cows in Mekelle, Ethiopia.

**Methods:**

An ambispective study design was employed, integrating retrospective data (June 2023–May 2024) and prospective observations (October 2024–May 2025). A total of 156 dairy farms were selected using stratified random sampling for the assessment of vaccination status and milk yield. Additionally, 56 lactating cows were selected for milk quality evaluation, with pre‐ and postvaccination comparisons. Milk composition analysis was conducted on a subset of 40 cows meeting strict inclusion criteria. Data were analyzed using R software (version 4.3.3).

**Major Findings:**

Vaccination coverage reached 78.85% in 2023/24 and 73.08% in 2024/25, mainly against anthrax, blackleg, and lumpy skin disease. Most farmers (80.71%) observed a short‐term decline in milk yield postvaccination, while 68.29% reported no long‐term yield change. Prior vaccination was a significant predictor of higher yield postvaccination (*β* = 2.93, *p* < 0.001). Laboratory results showed significant reductions in bacterial load (*p* = 0.0019) and significant increases in milk fat (*p* = 0.0218), protein (*p* = 0.0123), SNF (*p* = 0.0056), lactose (*p* = 0.0023), and ash (*p* = 0.0088).

**Conclusions:**

Cattle vaccination improved milk quality while causing only temporary yield declines. Strengthening regular vaccination programs and farmer awareness could enhance milk safety, nutritional value, and farm profitability, supporting Ethiopia’s broader food security and One Health goals.

## 1. Introduction

The dairy industry plays a vital role in Ethiopia’s agricultural economy, contributing significantly to household nutrition, income, and livelihoods. Despite the country’s large cattle population, national milk productivity remains far below potential, largely due to the high prevalence of infectious diseases and insufficient preventive health interventions [1, 2]. Vaccination is a key component of livestock health management and is known to reduce morbidity, mortality, and production losses [3]. However, in many low‐ and middle‐income countries, including Ethiopia, the direct effects of vaccination on milk yield and milk composition are not well understood under routine farm conditions.

Ethiopia possesses substantial potential for dairy development because of its favorable agro‐ecology and an estimated 59.5 million cattle. Nevertheless, milk self‐sufficiency remains low. The average dairy cow produces only 1.37 L per day, amounting to a national production of roughly 3.1 billion liters annually [2]. Infectious diseases remain a major constraint, largely due to inadequate preventive and control measures [4]. As a preventive strategy, vaccination is widely recognized as one of the most effective tools for limiting livestock disease. In dairy systems, where animal health directly influences productivity and profitability, immunization helps sustain milk yield, improve reproductive performance, and reduce economic losses associated with disease outbreaks. Vaccination also supports key production indicators such as lactation length, growth rates, breeding success, and conception rates [5].

Vaccines function by stimulating a controlled immune response similar to natural infection. This process temporarily triggers physiological and metabolic changes that may affect dairy cow performance. Following antigen exposure, inflammatory cytokines including interleukin‐1β, tumor necrosis factor‐α, and interferon‐γ can increase body temperature, reduce feed intake, and shift nutrient allocation [6, 7]. These immunometabolic adjustments may lead to short‐term reductions in milk production as nutrients are redirected toward immune demands rather than milk synthesis. In contrast, effective immunization lowers the incidence of clinical and subclinical diseases, helping reduce inflammation and metabolic stress over the long term. Improved herd health following vaccination has been associated with enhanced udder health, reduced bacterial contamination, improved milk hygienic quality, and maintenance or improvement of milk constituents such as fat, protein, lactose, and solids‐not‐fat (SNF) [5, 8, 9].

Despite the biological rationale for these mechanisms, there is minimal field‐based evidence from African dairy systems. Existing studies have largely been conducted under controlled conditions, relied on single vaccines, or originated from high‐income production environments [10, 11]. Little is known about the effects of Ethiopia’s routine multiantigen vaccination programs, particularly those targeting anthrax, blackleg, and lumpy skin disease on milk yield and composition in crossbred dairy cows managed under medium‐scale production systems, such as those in Tigray.

The Ethiopian dairy sector continues to underperform, with annual milk production growing at only 1.2%, which is well below the national population growth rate of 3% [12]. Milk is a nutritionally dense food whose composition influences both its economic value and consumer acceptability. Changes in milk components directly affect its quality and suitability for processing [13]. Consequently, understanding both milk yield and quality is essential for assessing dairy sector performance.

Previous studies have reported variable effects of vaccination on dairy production parameters. While several authors observed temporary reductions in milk yield immediately after vaccination due to immune activation and physiological stress [6, 7, 14], others documented improvements or maintenance of production performance following successful disease prevention [8]. Similarly, reports on milk composition have been inconsistent, with some studies finding minimal changes and others reporting alterations in fat, protein, lactose, and other quality indicators after vaccination or disease‐control interventions [9, 11, 15].

Given the limited mechanistic and field‐based evidence in Ethiopia and the heightened need for data to guide vaccination strategies in postconflict Tigray, there remains a substantial knowledge gap regarding vaccine coverage and the influence of common cattle vaccines on milk yield and composition under practical farm conditions. By integrating farm vaccination records, farmer‐reported outcomes, and laboratory‐based milk analyses, this study aims to generate foundational evidence that can inform future controlled research and support One Health–oriented dairy development efforts.

Therefore, the objectives of this study were as follows:

• To determine the percentage of cattle vaccinated against prevalent diseases on medium‐scale dairy farms
• To determine the impact of vaccination on milk quantity (total yield)
• To determine the impact of vaccination on milk quality parameters

## 2. Methods

### 2.1. Description of the Study Area

The study was conducted in Mekelle, the capital city of the Tigray regional state of Ethiopia. Mekelle is located 783 km north of Addis Ababa at geographic coordinates of 13°31′ N and 39°29′ *E*. The area has a semiarid climate with annual rainfall ranging from 530 to 714 mm, primarily during July–September. The average annual temperature ranges from < 15°C in highland areas to > 25°C in lowland areas. The city has an estimated cattle population of 36,516, with approximately 1565 dairy farms, including 220 medium‐scale farms.

### 2.2. Study Design

An ambispective observational study design integrating retrospective data (June 2023–May 24) and prospective observations (October 2024–May 2025) was employed. Data were collected at a single time point using structured questionnaires, farm records, and laboratory analyses. The retrospective component captured vaccination status and milk yield data from the previous year, while the prospective component assessed current vaccination practices and associated outcomes.

### 2.3. Study Population

The study population included dairy farmers and dairy cows of local zebu breeds, Holstein‐Friesian, Jersey, and their crossbreeds, which are commonly managed under medium‐scale dairy systems in the study area. The inclusion criteria for this study consisted of medium‐scale dairy farms in Mekelle, Tigray, which had a minimum number of 6 dairy cows and a maximum number of 30 dairy cows [16] and were actively engaged in milk production. Additionally, farm owners were willing to participate in the interview process. The exclusion criteria were farms that had fewer than 5 or more than 30 dairy cows, did not have lactating cows, or were not engaged in milk production at that time. Farms whose owners were unwilling to participate in interviews were also excluded.

### 2.4. Sample Size Determination

The sample size was determined by Cochran’s formula for finite populations [17]. Given that population size, *N* = 220; expected proportion, *p* = 0.5; margin of error, *E* = 0.05; confidence level, *Z* = 1.96, the initial sample size *n* = *Z* ^2^ × *p* × [1 − *p*]/*E* ^2^ = 384.16. Then, the adjusted sample size for the finite population *N* (220) was calculated as ≈140; the formula for *n*_adjusted_ = *n*/1 + ((*n* − 1)/*N*) ≈ 140.

To account for an expected 90% response rate, the sample size was further adjusted to ensure that the target was met after a 10% nonresponse rate was considered. Thus, the final sample size was calculated as: (140/(1 − 0.1)) = (140/0.90) ≈ 156. Hence, 156 dairy farms were included for vaccination status and milk yield assessment through structured questionnaires, and from these farms, 56 lactating cows were selected for detailed laboratory‐based milk quality evaluation, including pre‐ and postvaccination comparisons.

### 2.5. Sampling Procedure

A stratified random sampling method was used to select 156 farms from the total 220 medium‐scale farms. Stratification was based on subcities (Semien, Ayder, Kedamay‐Weyane, Hadinet, Adihaki, Hawelti, and Qwiha) to ensure representativeness. Within each stratum, farms were randomly selected proportionally.

Milk sampling was conducted purposively on 10 farms selected based on accessibility, willingness to participate, and availability of complete vaccination records. From these farms, 56 lactating cows were selected for microbiological analysis using systematic random sampling.

### 2.6. Data Collection

A semistructured questionnaire was developed in three stages. First, the questions were adapted from published instruments on dairy herd vaccination and milk production in low‐ and middle‐income countries, ensuring alignment with the study objectives. Second, a panel of five veterinary and dairy science experts reviewed the draft for clarity, coverage, and scientific relevance. Each item was rated for relevance on a 1–4 scale, and item content validity index (I‐CVI) values were calculated. Items with an I‐CVI < 0.78 were revised or removed, and the scale content validity index (S‐CVI/Ave) was computed. All the items achieved acceptable content validity (I‐CVI ≥ 0.80; S‐CVI/Ave = 0.92). Third, a pilot test on 15 nonsampled farms was performed to assess the clarity, flow, and completion time. Multi‐item constructs were tested for internal consistency (Cronbach’s *α* = 0.82) and test–retest reliability in a subsample (intraclass correlation coefficient = 0.87). The finalized questionnaire covered farm demographics, herd characteristics, vaccination history, milk yield, and handling practices, helping in gathering information on previous‐ and current‐year vaccination status and milk yield before and after vaccination. The final version is provided in Supporting File as Questionnaire Tool (available here).

### 2.7. Milk Sampling and Laboratory Analyses

Milk samples (*n* = 56) were collected for microbiological analysis. For milk composition analysis, a subset of 40 samples was selected from the 56 collected samples based on sample integrity, completeness of paired pre‐ and postvaccination data, and absence of contamination or handling errors. This ensured analytical reliability and comparability of compositional parameters. Each milk sample was collected aseptically according to National Mastitis Council [18] procedures. Teats were cleaned with water, dried via disposable towels, and disinfected with 70% ethanol. The first streams were discarded, and midstream composite samples (all quarters) were collected into sterile 50‐mL vials. Each sample was labeled with farm and cow identification, date, and time.

Milk yield data were obtained from 156 dairy farms through questionnaire interviews and farm records, whereas 56 paired milk samples were collected for microbiological analysis and 40 paired samples were analyzed for milk composition. Total plate count (TPC) assays were performed in duplicate, while milk composition parameters were measured according to the manufacturer’s standard operating procedure using a Lactoscan SP analyzer. Paired milk samples were collected from each cow 1 week before vaccination and 3 months after vaccination.

The samples were placed in a cold box (1°C–4°C) immediately after collection and transported to Mekelle University, College of Veterinary Science, Veterinary Microbiology Laboratory, within 24 h for TPC and in the Dairy Laboratory at the College of Dryland Agriculture and Natural Resources of Mekelle University for other milk quality parameters. If same‐day processing was not possible, the samples were refrigerated at 4°C and analyzed within 48 h.

#### 2.7.1. Microbiological Analysis

Microbiological analysis was performed on all 56 milk samples using TPC methods following ISO 4833‐1:2013 standards [19, 20]. The TPC for 56 milk samples was determined following ISO 4833‐1:2013 procedures, as also applied in similar milk quality assessments by Fadillah et al. [21]. Serial 10‐fold dilutions were prepared using sterile peptone saline. Appropriate dilutions were plated in duplicate via the pour‐plate method on plate count agar and incubated aerobically at 30°C for 72 h. Plates with 15–300 colonies were counted, and the results are expressed as colony‐forming units per milliliter (CFU/mL). The results were categorized according to the Ethiopian standard (ES 3460:2009) [31]: very good (< 2 × 10^5^ CFU/mL), good (2 × 10^5^ − 1 × 10^6^ CFU/mL), bad (1 × 10^6^ − 2 × 10^6^ CFU/mL), and very bad (> 2 × 10^6^ CFU/mL).

#### 2.7.2. Milk Composition Analysis

Milk composition analysis was conducted on a subset of 40 cows selected from the 56 sampled animals based on complete paired pre‐ and postvaccination data, proper sample handling, and absence of contamination, ensuring analytical accuracy and comparability. Milk constituents, including fat, protein, SNF, lactose, and ash, were analyzed via a Lactoscan SP ultrasonic milk analyzer [23], which was calibrated before each batch using the manufacturer‐provided standards. In detail, the milk sample was homogenized by turning down the sample bottle, and the lactoscan was cleaned with lactoscan diluted daily in distilled water at a ratio of 1:10. Then, a 10‐mL milk sample was placed in the sample tube and placed in the sample holder in the recess position. After that, the starting button was activated, the analyzer sucked the milk, made measurements, and returned the milk in the sample tube, and the digital indicator showed the specified results.

### 2.8. Vaccine Details and Manipulations

Vaccination data were collected from farm records and interviews with farm owners. The information recorded included the vaccine trade name, target disease (anthrax, blackleg, lumpy skin disease), manufacturer, batch number, expiration date, route of administration, dose, vaccination date, and person administering the vaccine (veterinarian or paravet). Farmers were also interviewed regarding any postvaccination reactions, such as fever, local swelling, reduced milk yield, or appetite loss (Supporting file, Questionnaire Tool).

### 2.9. Data Analysis

The data collected were processed and analyzed via R statistical software (version 4.3.3). Descriptive statistics, including frequency counts and percentages, were applied to summarize vaccination coverage and observed milk yield changes. The Stuart–Maxwell test was used to determine significant shifts in bacterial load between pre‐ and postvaccination samples. For nutritional quality parameters (fat, protein, SNF, lactose, and ash), McNemar’s test was conducted to assess paired changes. Chi‐square tests and Fisher’s exact test were utilized to explore the relationships between disease outbreaks and milk yield decline, as well as between vaccination status and changes in income from milk sales. Ordinal logistic regression was employed to identify key predictors of postvaccination milk yield, with significant variables including prior vaccination status, breed, and feeding changes. Graphical outputs such as bar charts and transition diagrams were used to visualize key trends and associations.

## 3. Results

Milk yield and vaccination data covered a 12‐month retrospective period (June 2023–May 2024), while prospective observations were conducted over an 8‐month period (October 2024–May 2025).

### 3.1. Vaccine Coverage Against Anthrax, Blackleg, and LSD

The results revealed the status of Anthrax, Blackleg, and LSD cattle vaccine coverage among medium‐scale dairy farms in Mekelle, Tigray, over two time periods: 2023/24 and 2024/25. Among the total farms surveyed, 78.85% reported that they had vaccinated their cows in the previous year. For the current year, 73.08% of the cows were vaccinated (Table 1).

**TABLE 1:** Proportion of vaccinated vs. unvaccinated cows in the previous and current years.

	Category	Frequency	Proportion (%)
Farms vaccinated their cows last year	No	33	21.15
	Yes	123	78.85

Farms vaccinated their cows in this year	No	42	26.92
	Yes	114	73.08

### 3.2. Impact of Vaccination on Milk Yield

**FIGURE 1:**
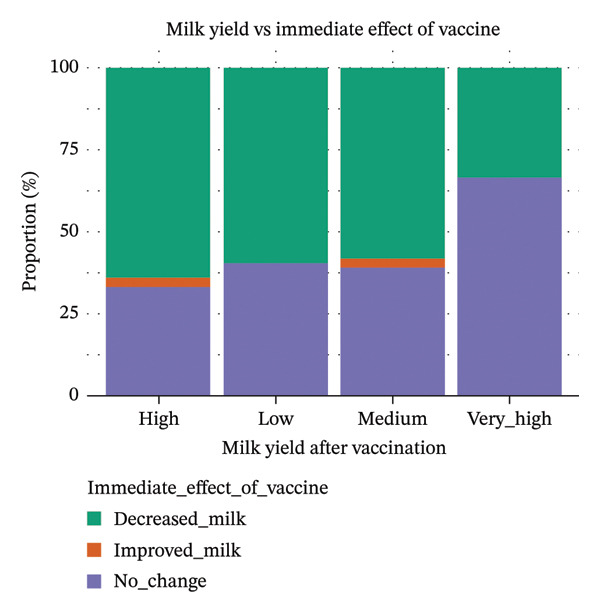
Graph of milk yield after vaccination vs the immediate effect of the vaccine.

The immediate, transient effect of vaccination on milk yield was assessed, and the results revealed that the majority of respondents (80.71%) reported a temporary decrease in milk yield immediately in the days following vaccination. When the overall change in milk yield after the vaccination period (beyond the immediate, transient effect) was evaluated, 68.29% of the respondents indicated that there was no change in milk yield, as shown in Table 2 and/or Figure 1.

**TABLE 2:** Vaccine impact on milk yield.

	Vaccination status	Category	Frequency	Proportion (%)
Immediate transient effect of vaccination on milk yield (short‐term effect)	Yes (this year)	Decreased	92	80.71
		No change	20	17.54
		Increased	2	1.75

Overall milk yield change after vaccination (long‐term effect)	Yes (previous year)	Decreased	6	4.88
		No change	84	68.29
		Increased	33	26.83

However, a highly significant association was found between current vaccination status and the immediate effect of vaccination on milk yield, as confirmed by Fisher’s exact test (*p* < 0.001), and the relationship between prior vaccination and the perceived change in milk yield postvaccination was also statistically significant (*χ* ^2^ = 32.67, *p* < 0.001), as shown in Table 3.

**TABLE 3:** Milk yield vs. related variable impact.

Analysis	Chi‐square (*χ* ^2^)	Degree of freedom (df)	*p* value
Vaccination last year × Change after vaccination	32.67	2	8.069244*e* − 08
Currently vaccinated × Immediate effect of vaccine (Fisher’s exact test)	—	—	<2.2*e* − 16
Vaccination last year × Milk production impact on income	19.54	2	5.714691*e* − 05
Milk yield × Disease occurrence in the farm	18.32	2	0.0001

### 3.3. Milk Yield vs Disease Outbreak

**FIGURE 2:**
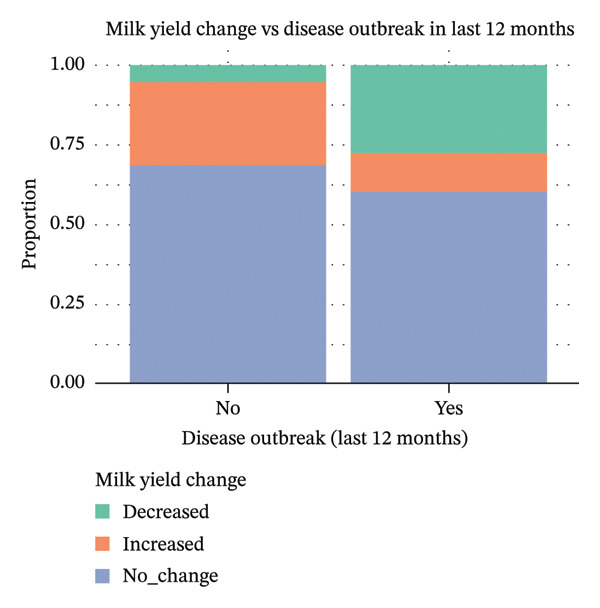
Milk yield vs disease outbreak (associated with vaccination).

A significant association was found between disease outbreaks in the past 12 months and changes in milk yield after vaccination. Farms with reported outbreaks were more likely to report decreased milk yield (*χ* ^2^ = 18.32, *p* = 0.0001) (Figure 2).

### 3.4. Milk Yield vs Economic Performance

There was a statistically significant relationship between milk yield changes and farm income. Farms that experienced improved or stable milk yield after vaccination were more likely to report increased income over the past year (*χ* ^2^ = 19.54, *p* < 0.001), as shown in Table 3. Additionally, ordinal logistic regression revealed that vaccination in the previous year was a significant predictor of higher milk yield after vaccination (*β* = 2.93, *p* < 0.001) (Table 4).

**TABLE 4:** Predictors affecting milk yield post‐vaccination.

Predictor	Estimate (*β*)	Std. error	*t* value	*p* value
Vaccinated in 2023/24 (yes)	2.93	0.580	5.04	< 0.001
Feed change in the last 12 months (yes)	1.01	0.444	2.27	< 0.05
Main breed (local)	−4.92	0.925	−5.32	< 0.001

### 3.5. Impact of Vaccination on Milk Quality

Microbiological analysis was conducted on all 56 collected milk samples, while compositional quality analysis was performed on a subset of 40 samples meeting quality control criteria.

#### 3.5.1. Bacterial Load

A significant reduction in bacterial contamination was observed after vaccination. The Stuart–Maxwell test yielded a chi‐square value of 14.9 (*p* value = 0.0019), indicating a statistically significant improvement (Table 3).

#### 3.5.2. Nutrient Quality Changes

McNemar’s test revealed significant improvements across all measured nutrients postvaccination; significant increases in fat (*p* value = 0.0218), protein (*p* value = 0.0123), SNF (*p* value = 0.0056), lactose (*p* value = 0.0023), and ash (*p* value = 0.0088) contents were recorded, as shown in Table 5.

**TABLE 5:** Impact of the vaccine on milk quality.

Milk quality parameters	Chi‐square value (*χ* ^2^)	Degree of freedom (df)	*p* value
Bacterial load	14.9	3	0.0019
Fat	5.2632	1	0.0218
Protein	6.2609	1	0.0123
SNF	7.6818	1	0.0056
Lactose	9.3333	1	0.0023
Ash	6.8571	1	0.0088

**FIGURE 3:**
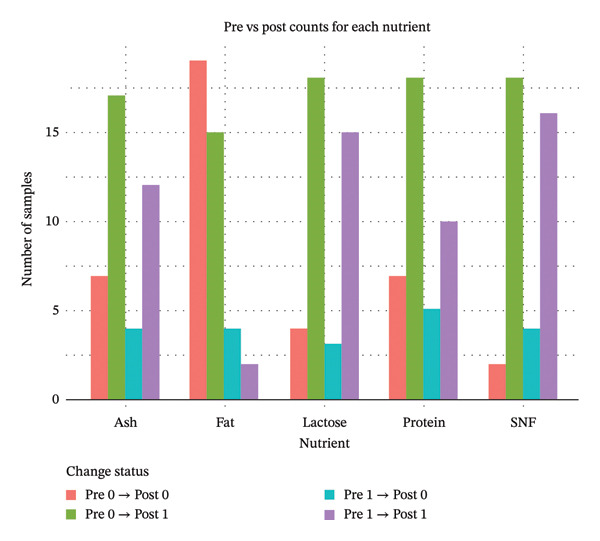
Transition of milk nutrient‐status indicators (fat, protein, SNF, lactose, and ash) before and after vaccination. *Note*. Pre 0 ⟶ post 0: stayed low/absent (no change—nutrient level remained low), pre 0 ⟶ post 1: increased (nutrient improved or increased after vaccination), pre 1 ⟶ post 0: decreased (nutrient dropped or worsened post‐intervention), and pre 1 ⟶ post 1: stayed high (no change—nutrient level remained high).

As shown in Figure 3, the milk samples that were very bad (> 2 × 106 CFU/mL bacteria), bad (1 × 10^6^ − 2 × 10^6^ CFU/mL), and good (2 × 10^5^ − 1 × 10^6^ CFU/mL) during the prevaccination period were all classified as very good (< 2 × 10^5^ CFU/mL) during the postvaccination period. The nutrient‐status transition analysis (Figure 3) showed that more samples shifted from low to high nutrient‐status categories (Pre 0 ⟶ Post 1) than from high to low categories (Pre 1 ⟶ Post 0). These findings are consistent with the statistical comparisons presented in Table 5, which demonstrated significant postvaccination improvements in fat (*p* = 0.0218), protein (*p* = 0.0123), SNF (*p* = 0.0056), lactose (*p* = 0.0023), and ash (*p* = 0.0088) contents.

## 4. Discussion

In the present study, of the total farms surveyed, 78.85% reported vaccinating their cows in 2023/24, whereas 73.08% reported vaccination in 2024/25. Although the majority of farms maintained vaccination practices across both years, there was a slight decrease in the proportion of vaccinated farms in that year. In both years, the proportion of vaccinated farms was greater than that of unvaccinated farms. This coverage was better than that reported by Sarker et al. [24] and Endalew and Wakene [4], who reported 44% Anthrax Vaccine coverage in 2020 in Bangladesh and 36% LSD vaccine coverage in 2019 in the Digelu‐tijo district in the Arsi zone.

A transient decrease in milk yield was reported by 80.71% of the respondents on the days immediately following vaccination, and a highly significant association was found between current vaccination status and the immediate effect of vaccination on milk yield, as confirmed by Fisher’s exact test (*p* < 0.001). This observation aligns with Abutarbush et al. [14], Schulze et al. [6], Katsoulos et al. [11], Krishnaswamy et al. [7], and Pramod et al. [25], who reported transient short‐term milk yield reductions in Jordan, Germany, northern Greece, Bengaluru (India), and Kerala (India), respectively. This reduction may be due to stress associated with restraint of the cow for vaccination, vaccine‐induced pain, and/or an inflammatory response that induces a reduced appetite and decreases nutrient availability, potentially lowering milk yield.

Beyond the transient decrease, the majority of respondents (68.29%) indicated no long‐term change in milk yield. The relationship between prior vaccination and the perceived change in milk yield postvaccination was statistically significant (*χ* ^2^ = 32.67, *p* < 0.001). This finding is in agreement with Schmitt‐Van et al. [8], who reported significant increases in milk yield after vaccination with a live double‐deleted bovine viral diarrhea virus (BVDV) vaccine in the United Kingdom and Italy. However, this finding contrasts with findings from Morgenstern and Klement [10], who reported no significant difference in milk yield between vaccinated and nonvaccinated cows with the live attenuated Neethling LSD vaccine in Israel. This discrepancy may be due to differences in vaccine type.

A significant association was found between disease outbreaks in the past 12 months and changes in milk yield after vaccination. Farms with reported disease outbreaks were more likely to report decreased milk yield. These findings are in agreement with those of Kiplagat et al. [26] and Vinitchaikul et al. [27], who reported a significant decrease in milk yield after the LSD outbreak in Kenya and Thailand.

A significant association was also found between milk yield changes and farm income. Farms that experienced improved or stable milk yield after vaccination were more likely to report increased income over the past year. This finding aligns with that of Ozturk et al. [28], who reported an improvement in farmer income after vaccination for foot and mouth disease (FMD) in Turkey.

One of the key findings of this study is that, in crossbred cows (Holstein Friesian X Zebu), vaccination against anthrax, blackleg, and LSD resulted in a significant decrease in the total bacterial count and a significant increase in the fat, protein, solid but not fat, lactose, and ash contents of milk at 3 months post‐vaccination. Milk samples that were very bad, bad, and good pre‐vaccination were all converted to very good milk post‐vaccination. Additionally, the fat, protein, SNF, lactose, and ash contents either remained high or improved after vaccination. These findings contrast with previous studies reporting no significant changes in milk fat, protein, lactose, or SNF following vaccination [11, 25], while other studies reported reductions in specific milk constituents, particularly protein content, following vaccination or disease‐control interventions [9, 15]. Conversely, Schmitt–van de Leemput et al. [8] reported improvements in productive performance following vaccination, supporting the possibility that improved animal health status may indirectly contribute to better milk quality outcomes.

In contrast, the present study employed combined vaccination and sampled milk 3 months post‐vaccination, potentially capturing chronic immune‐metabolic effects. Compared with single‐antigen vaccines, combined vaccines may stimulate a stronger immune response. Additionally, factors such as breed, diet, individual animal genetics, stage of lactation, management and seasonality, as well as the interactions between them, affect milk composition [29]. Therefore, comparing milk from different animals, as in earlier studies, may mask true vaccine effects.

The difference in sample sizes between microbiological (*n* = 56) and compositional analyses (*n* = 40) was due to strict inclusion criteria for compositional testing, including complete paired sampling, proper storage conditions, and absence of contamination. This approach ensured the validity and reliability of milk composition measurements.

Despite providing valuable insights, this study has several limitations. The first limitation is the lack of a controlled comparison group of unvaccinated farms, which limits the ability to definitively attribute changes in milk yield and quality to vaccination alone. Additionally, much of the data on milk yield and postvaccination effects were based on farmers’ self‐reported perceptions rather than direct, objective measurements, which may introduce recall or response bias.

## 5. Conclusions and Recommendations

This study assessed cattle vaccination coverage and its effects on milk yield and quality on medium‐scale dairy farms in Mekelle, Tigray. Vaccination coverage against anthrax, blackleg, and lumpy skin diseases decreased slightly from 78.85% in 2023/24 to 73.08% in 2024/25. Although most farmers (80.71%) reported a short‐term decline in milk yield immediately after vaccination, the majority (68.29%) reported no long‐term changes. Importantly, prior vaccination was significantly associated with farmers’ perceptions of milk yield outcomes. Laboratory analyses demonstrated that vaccination significantly improved milk quality, with reductions in bacterial load and increases in fat, protein, SNF, lactose, and ash contents 3 months post‐vaccination. Overall, vaccination was linked to transient effects on yield but consistent benefits in terms of milk quality. These findings underscore the contribution of livestock vaccination to food system resilience, improved nutritional quality of milk, and ultimately better public health outcomes in Ethiopia.

### 5.1. Recommendations

1. Routine vaccination programs should be strengthened to ensure broad and consistent coverage across dairy farms, including targeted support for smallholder farmers.
2. Provide farmer training on the importance of timely and combined vaccinations to sustain herd health, milk quality, and overall nutritional security.
3. Supportive management practices, such as improved feeding and stress reduction strategies, should be promoted to minimize short‐term declines in milk yield following vaccination.
4. Vaccination strategies should be integrated into livestock and nutrition‐sensitive agricultural policies to increase food system resilience and public health.
5. Further research should be conducted to examine the long‐term effects of vaccination on milk yield, milk composition, and nutritional outcomes across diverse production systems, breeds, and geographic settings.

## Author Contributions

Tadelech Yilma Sisay conceived and designed the study, coordinated data collection, performed statistical analyses, interpretation of laboratory findings, preparation of tables and figures, drafted the manuscript, and critically revised the manuscript for important intellectual content.

Haftom Yirga Tsegay, Mastewal Addisu Kitambo, Mebrahtu Weldegebriel Hailu, and Kidist Derbew Gebru contributed to study design, supervised data collection in the field, and conducted laboratory analyses of milk quality parameters.

## Funding

This study was supported by small‐scale funding from NORAD with award number of RADO/EXTERNAL/SS0027/2024.

## Ethics Statement

Farmers provided verbal (oral) consent prior to participation. No human subjects were directly involved, and no ethics committee approval was needed. All procedures were conducted in a manner that ensured no harm or stress to the animals. The sampling process was noninvasive and adhered to ethical standards for animal welfare and research involving livestock.

## Consent

The authors have nothing to report.

## Conflicts of Interest

The authors declare no conflicts of interest.

## Supporting Information

Additional supporting information can be found online in the Supporting Information section.

## Supporting information


**Supporting Information** Supporting 1. Questionnaire Tool: Supporting 1 contains the structured questionnaire used for data collection from dairy farm owners/managers and veterinarians during both the retrospective and prospective phases of the study. The questionnaire was developed based on the study objectives and relevant published instruments, followed by expert review and pilot testing to ensure content validity and reliability. It comprises five sections covering (i) general farm information, (ii) retrospective vaccination history and milk production, (iii) prospective vaccination and milk production data, (iv) potential confounding factors related to feeding and herd management, and (v) additional observations regarding farmers’ perceptions of vaccination. The questionnaire served as the primary tool for collecting information on vaccination coverage, herd characteristics, milk yield, milk quality, and management practices. All information was collected confidentially and used exclusively for research purposes.

## Data Availability

Data supporting the findings are available from the corresponding author upon reasonable request.

## References

[1] Tadesse A. and Negash N. , Prevalence and Economic Connotation of Bovine and Caprine Hydatidosis at Abergele International Export Slaughterhouse, Mekele, Tigray Region, Ethiopian Veterinary Journal. (2022) 26, no. 2, 38–56, 10.4314/evj.v26i2.3.

[2] Dekebo D. and Kebede I. A. , Review on Dairy Cattle Production in Ethiopia, Mathews Journal of Veterinary Science. (2023) 7, no. 4, 1–17.

[3] Robi D. T. , Bogale A. , Temteme S. , Aleme M. , and Urge B. , Evaluation of Livestock Farmers’ Knowledge, Attitudes and Practices Regarding the Use of Veterinary Vaccines in Southwest Ethiopia, Veterinary Medicine and Science. (2023) 9, no. 6, 2871–2884, 10.1002/vms3.1290. 37788141 PMC10650347

[4] Endalew M. A. and Wakene F. S. , Retrospective Study on Livestock Vaccine Coverage and Trends in Digelu-Tijo District, Arsi Zone, International Journal of Agricultural Extension. (2020) 8, no. 3, 219–224, 10.33687/008.03.3394.

[5] Aslam M. A. , Rafique M. N. , Rauf U. et al., Alvi M. A. , Rashid M. , Zafar M. A. , Mughal M. A. S. , and Toor S. I. , Immunization: Role of Vaccines in Preventing Disease Challenges at Dairy Farms, Complementary and Alternative Medicine: Immunization/Vaccinology, 2024, Unique Scientific Publishers, Faisalabad, Pakistan, 365–372.

[6] Schulze L. S.-Ch. , Borchardt S. , Ouellet V. , and Heuwieser W. , Effect of a Phase I *Coxiella burnetii* Inactivated Vaccine on Body Temperature and Milk Yield in Dairy Cows, Journal of Dairy Science. (2016) 99, no. 1, 541–550, 10.3168/jds.2015-9628. 26547657

[7] Krishnaswamy N. , Jeyakumar S. , Selvan R. P. T. et al., Short-Term Effect of Foot-and-Mouth Disease (FMD) Vaccination on the Milk Yield in the Deoni and Crossbred Cows, Tropical Animal Health and Production. (2021) 53, no. 2, 10.1007/s11250-021-02653-y. 33745013

[8] Schmitt–van de Leemput E. , Metcalfe L. V. A. , Caldow G. , Walz P. H. , and Guidarini C. , Comparison of Milk Production of Dairy Cows Vaccinated With a Live Double Deleted BVDV Vaccine and Nonvaccinated Dairy Cows Cohabitating in Commercial Herds Endemically Infected With BVD Virus, PLoS One. (2020) 15, no. 10, 10.1371/journal.pone.0240113. PMC7529212 33002072

[9] Pintos C. G. and Menchaca A. , Effect of Vaccination Against Foot-and-Mouth Disease on Milk Yield in Dairy Cows, Research Square. (2024) . 10.1016/j.vas.2026.100683 PMC13213856 42211229

[10] Morgenstern M. and Klement E. , The Effect of Vaccination With Live Attenuated Neethling Lumpy Skin Disease Vaccine on Milk Production and Mortality: An Analysis of 77 Dairy Farms in Israel, Vaccines. (2020) 8, no. 2, 10.3390/vaccines8020324. PMC7350216 32575395

[11] Katsoulos D. , Chaintoutis S. C. , Dovas C. I. et al., Investigation on the Incidence of Adverse Reactions, Viraemia and Hematological Changes Following Field Immunization of Cattle Using a Live Attenuated Vaccine Against Lumpy Skin Disease, Transboundary and Emerging Diseases. (2018) 65, no. 1, 174–185, 10.1111/tbed.12646. 28391652

[12] Mihret T. , Mitku F. , and Guadu T. , Dairy Farming and Its Economic Importance in Ethiopia: A Review, World Journal of Dairy & Food Sciences. (2017) 12, no. 1, 42–51.

[13] Desyibelew W. and Wondifraw Z. , Evaluation of Milk Composition in Zebu × HF Crossbred Dairy Cows in Different Seasons and Stage of Lactations in Amanuel Town, Ethiopia, Journal of Agricultural Science and Food Research. (2019) 10, no. 1, 10.35248/2593-9173.19.10.255.

[14] Abutarbush S. M. , Hananeh W. M. , Ramadan W. et al., Adverse Reactions to Field Vaccination Against Lumpy Skin Disease in Jordan, Transboundary and emerging diseases. (2016) 63, no. 2, e213–e219, 10.1111/tbed.12257. 25098267

[15] Angelova T. , Yordanova D. , Krastanov J. et al., Quantitative and Qualitative Changes in Milk Yield and Cheese-Making Properties of Milk in Cows Vaccinated Against Lumpy Skin Disease, Macedonian Journal of Animal Science. (2018) 8, no. 2, 89–95, 10.54865/mjas1882089a.

[16] Kidane A. B. , Delesa K. E. , Mummed Y. Y. , and Tadesse M. , Reproductive and Productive Performance of Holstein Friesian and Crossbreed Dairy Cattle at Large, Medium and Small Scale Dairy Farms in Ethiopia, International Journal of Advanced Research in Biological Sciences. (2019) 6, 15–29.

[17] Cochran W. G. , Some Methods for Strengthening the Common Χ^2^ Tests, Biometrics. (1954) 10, no. 4, 417–451, 10.2307/3001616.

[18] National Mastitis Council (NMC) , Procedures for Collecting Milk Samples, 2004, National Mastitis Council, Madison, WI, USA, https://www.nmconline.org/wp-content/uploads/2016/09/Procedures-for-Collecting-Milk-Samples.pdf.

[19] International Organization for Standardization , ISO 4833-1:2013, Microbiology of the Food Chain—Horizontal Method for the Enumeration of Microorganisms: Part 1: Colony Count at 30°C by the Pour Plate Technique, 2013, ISO, Geneva, Switzerland.

[20] U.S. Food and Drug Administration , Bacteriological Analytical Manual (BAM) Chapter 3: Aerobic Plate Count–May 2025 Edition, 2025, FDA, Silver Spring, MD.

[21] Fadillah A. P. B. H. , Poetri O. N. , Hogeveen H. , Slijper T. , Pisestyani H. , and Schukken Y. H. , Evaluation of Factors Associated With Bulk Milk Somatic Cell Count and Total Plate Count in Indonesian Smallholder Dairy Farms, Frontiers in Veterinary Science. (2023) 10, 10.3389/fvets.2023.1280264. PMC10713723 38089701

[22] Ethiopian Standards Agency , Unprocessed Whole/Raw Cow Milk Specification (2Nd, ES: 3460:2009). Addis Ababa, Ethiopia, 2009.

[23] Milkotronic Ltd , Lactoscan SP Ultrasonic Milk Analyzer: Operation Manual. Stara Zagora, 2014, Milkotronic Ltd, Bulgaria, https://www.milkotronic.com/pdfs/SPbooklet.pdf.

[24] Sarker M. S. , El Zowalaty M. E. , Shahid M. A. et al., Maximization of Livestock Anthrax Vaccination Coverage in Bangladesh: An Alternative Approach, Vaccines. (2020) 8, no. 3, 10.3390/vaccines8030435. PMC7563472 32759647

[25] Pramod S. , ThirupathyVenkatachalapathy R. , Sahib L. , and Becha B. , Influence of FMD Vaccination Stress on Milk Production in Crossbred Dairy Cattle of Kerala, Indian Journal of Dairy Science. (2021) 74, no. 1, 95–99, 10.33785/ijds.2021.v74i01.013.

[26] Kiplagat S. K. , Kitala P. M. , Onono J. O. , Beard P. M. , and Lyons N. A. , Risk Factors for Outbreaks of Lumpy Skin Disease and the Economic Impact in Cattle Farms of Nakuru County, Kenya, Frontiers in Veterinary Science. (2020) 7, 10.3389/fvets.2020.00259. PMC7274042 32548130

[27] Vinitchaikul P. , Punyapornwithaya V. , Seesupa S. et al., The First Study on the Impact of Lumpy Skin Disease Outbreaks on Monthly Milk Production on Dairy Farms in Khon Kaen, Thailand, Veterinary World. (2023) 16, no. 4, 687–692, 10.14202/vetworld.2023.687-692. 37235156 PMC10206973

[28] Ozturk N. , Kocak O. , and Vosough Ahmadi B. , Economic Analysis of Increasing Foot-and-Mouth Disease Vaccination Frequency: The Case of the Biannual Mass Vaccination Strategy, Frontiers in Veterinary Science. (2020) 7, 10.3389/fvets.2020.557190. PMC7597382 33195530

[29] Hermiz H. N. and Hadad J. M. , Factors Affecting and Estimates of Repeatability for Milk Production and Composition Traits in Several Breeds of Dairy Cattle, Indian Journal of Animal Sciences. (2020) 90, no. 3, 129–133, 10.56093/ijans.v90i3.102534.

