# Supplementary material for: Pre‐ and Postvaccination Effects on Milk Yield and Quality Among Dairy Farms and Lactating Cows in Mekelle, Ethiopia: An Ambispective Study
Source: Vet Med Int. 2026 Jul 19;2026:2417101. doi: 10.1155/vmi/2417101 (PMC13382153; doi:10.1155/vmi/2417101)
Supplement: Supplementary file 1 — Supporting Information Supporting 1. Questionnaire Tool: Supporting 1 contains the structured questionnaire used for data collection from dairy farm owners/managers and veterinarians during both the retrospective and prospective phases of the study. The questionnaire was developed based on the study objectives and relevant published instruments, followed by expert review and pilot testing to ensure content validity and reliability. It comprises five sections covering (i) general farm information, (ii) retrospective vaccination history and milk production, (iii) prospective vaccination and milk production data, (iv) potential confounding factors related to feeding and herd management, and (v) additional observations regarding farmers’ perceptions of vaccination. The questionnaire served as the primary tool for collecting information on vaccination coverage, herd characteristics, milk yield, milk quality, and management practices. All information was collected confidentially and used exclusively for research purposes. [file VMI-2026-2417101-s001.docx]

# SUPPLEMENTARY MATERIALS

Questionnaire Tool presents the structured questionnaire used in this study. The questionnaire was administered to dairy farm owners/managers and veterinarians to collect data on farm characteristics, herd vaccination status, vaccination coverage, milk yield, milk quality testing practices, and herd management. It includes both retrospective information from the previous 12 months and prospective data collected during the study period, as well as information on post-vaccination reactions and potential confounding factors such as feeding practices and herd health management. All data were collected confidentially and used exclusively for research purposes.

## 1.1. Questionnaire Tool

Includes the structured questionnaire to be completed by farm owners/managers and veterinarians and used to gather data on herd vaccination status, farm demographics, milk yield, and related management practices. All the responses are confidential and used solely for research purposes.

**Section 1: General Farm Information**

1. Farm Name: ____________________________

2. Location (Sub-City/Woreda): ___________________________

3. Farm size (number of cattle): ___________________________

4. Type of dairy farming: ☐ Intensive ☐ Extensive ☐ Mixed

5. Main Dairy Breed(s): ☐ Holstein ☐ Jersey ☐ Crossbred ☐ Local Breed

**Section 2: Retrospective Data on Vaccination and Milk Production (Past 12 Months)**

*Vaccination history*

| Vaccine Name | Target Disease | Manufac-turer | Batch/Lot No. | Expiry Date | Route (SC/  IM) | Dose (mL) | Date Administered | Administrator |
| --- | --- | --- | --- | --- | --- | --- | --- | --- |
|  |  |  |  |  |  |  |  |  |

8. Percentage of herd vaccinated: ☐ <25% ☐ 25–50% ☐ 51–75% ☐ >75%

9. Frequency of vaccination: ☐ Annually ☐ Semiannually ☐ Quarterly ☐ Other: ____________

10. Postvaccination reactions observed (check all that apply): ☐ Fever ☐ Swelling at the injection site ☐ Loss of appetite ☐ Decrease in milk yield ☐ No visible reaction ☐ Other: ____________

**Milk Production and Quality (Past 12 Months)**

11. Average daily milk yield per cow before vaccination (liters): ____________

12. Average daily milk yield per cow after vaccination (liters): ____________

13. Change in milk yield after vaccination: ☐ Increased ☐ Decreased ☐ No change

14. Do you conduct regular milk quality testing? ☐ Yes ☐ No

15. If yes, which parameters are tested? ☐ Fat content ☐ Protein ☐ Bacterial load ☐ Other: ____________

**Section 3: Prospective Data Collection (Current Study Period)**

*Vaccination Coverage (Current Year)*

| Vaccine Name | Target Disease | Manufact-urer | Batch/  Lot No. | Expiry Date | Route (SC/IM) | Dose (mL) | Date Administered | Administrator |
| --- | --- | --- | --- | --- | --- | --- | --- | --- |
|  |  |  |  |  |  |  |  |  |

19. Any postvaccination reactions observed (within 7 days)? ☐ Fever ☐ Swelling ☐ Reduced milk yield ☐ Anorexia ☐ None ☐ Other: ____________

**Milk Production and Quality (Current Study Period)**

20. Current average daily milk yield per cow (liters): ____________

21. Immediate change in milk yield within days following vaccination: ☐ Increased ☐ Decreased ☐ No change

22. Was milk quality tested this month? ☐ Yes ☐ No

23. If yes, what were the results? ☐ Increase in fat ☐ Increase in protein ☐ Reduction in bacterial load ☐ Other: ____________

**Section 4: Confounding Factors (Historical and Current)**

Feeding and Nutrition

24. Main feeding practice: ☐ Grazing ☐ Cut and carry ☐ Commercial feed ☐ Mixed (grazing + supplementary)

25. Do you provide supplementary feed? ☐ Yes ☐ No

26. If yes, what type(s)? ☐ Hay ☐ Straw ☐ Silage ☐ Concentrates ☐ Other: ____________

27. Has the feed type changed in the past 12 months? ☐ Yes ☐ No

*Herd Management and Health*

28. Frequency of veterinary health checks: ☐ Monthly ☐ Quarterly ☐ Yearly ☐ Only when sick

29. Major diseases observed in the past 12 months: ☐ Mastitis ☐ FMD ☐ Respiratory diseases ☐ Other: ____________

**Section 5: Additional Observations (Optional)**

30. Farmer’s perception of the effects of vaccination benefits on milk yield and quality: ________________________________

31. Suggestions for improving vaccination programs in your area: ________________________________
